# Agreement between fat-free mass from bioelectrical impedance analysis and dual-energy X-ray absorptiometry and their use in estimating resting metabolic rate in resistance-trained men

**DOI:** 10.1080/15502783.2024.2357319

**Published:** 2024-06-28

**Authors:** Alex S. Ribeiro, Sandro L. Sofiati, Witalo Kassiano, Diogo V. Martinho, Matheus A. Nascimento, Ademar Avelar, Michele C. C. Trindade, Jerry L. Mayhew, Edilson S. Cyrino

**Affiliations:** a University of Coimbra, Research Unit for Sport and Physical Activity, Faculty of Sport Sciences and Physical Education, Coimbra, Portugal; b University Pitágoras, UNOPAR, Londrina, Brazil; c State University of Londrina, Metabolism, Nutrition, and Exercise Laboratory, Londrina, Brazil; d Paraná State University (UNESPAR), Department of Physical Education, Paranavaí, Brazil; e State University of Maringá, Department of Physical Education, Maringá, Brazil; f Truman State University, Exercise Science Department, Kirksville, USA

**Keywords:** Resting metabolic rate, resistance training, bioelectrical impedance, dual-energy X-ray absorptiometry, prediction

## Abstract

**Background:**

This study aimed to determine the agreement between fat-free mass (FFM) estimates from bioelectrical impedance analysis (BIA) and dual-energy X-ray absorptiometry (DXA) and their use in estimating resting metabolic rate (RMR) in men undergoing resistance training.

**Methods:**

Thirty healthy resistance-trained men (22.7 ± 4.4 years, 70.0 ± 8.7 kg, 174.6 ± 6.7 cm, and 22.9 ± 2.3 kg/m^2^) were evaluated. The equation developed by Tinsley et al. (RMR = 25.9 × fat-free mass [FFM] + 284) was adopted to calculate the RMR. DXA was used as the reference method for FFM.

**Results:**

Furthermore, FFM was also estimated by BIA using a spectral device. No significant difference (*p* > 0.05) was observed between DXA (1884.2 ± 145.5 kcal) and BIA (1849.4 ± 167.7 kcal) to estimate RMR. A positive and significant correlation (*r* = 0.89, *p* < 0.05) was observed between DXA and BIA estimates of RMR. The mean difference between methods indicated that BIA presented a bias of −34.8 kcal.

**Conclusion:**

These findings suggest that using FFM derived from DXA or BIA results in similar RMR estimates in resistance-trained men.

## 1. Introduction

Estimating resting metabolic rate (RMR) is essential for developing strategies to induce changes in body composition, such as increasing skeletal muscle mass and reducing fat mass. Regarding the potential for muscle growth, an energy surplus is necessary to optimize muscle hypertrophy since a hypercaloric diet creates a favorable metabolic environment for muscle development [1,2]. In contrast, according to the first law of thermodynamics, achieving a negative energy balance is necessary to reduce body fat levels [3]. Therefore, knowledge of total daily energy expenditure is required to stipulate a hypercaloric or hypocaloric diet to promote either a caloric surplus or deficit.

Daily energy expenditure includes RMR, amount of physical activity and the thermic effect of food. RMR corresponds to the daily energy required to meet the energy expenditure related to the biological work of various organ systems. The RMR is , typically, the most significant contributor to energy expenditure and is equivalent to approximately 60–75% of total energy output [4]. To accurately quantify RMR, it is necessary to use laboratory techniques based on indirect calorimetry, which requires an accurate analysis of respiratory gases. However, a strict protocol in a laboratory environment and the availability of expensive equipment are necessary, which makes the practical application of indirect calorimetry infeasible. Therefore, prediction equations are the most suitable option for estimating RMR in a professional practical [5,6].

Most prediction equations developed to estimate RMR consider body mass as a primary variable for calculation and were obtained in samples of untrained individuals [7]. This can lead to errors for trained subjects owing to the difference in caloric expenditure of various body tissues since muscles expend almost three times more calories than adipose tissue [8]. Therefore, the difference between body mass components might impact RMR since two individuals with the same body mass but different relative body fat and skeletal muscle mass may present a sizable difference in RMR. Energy expenditure may be greater for leaner individuals than those with the same body weight but greater fat mass. Flack et al. [7] observed a greater underestimation of RMR in those with higher FFM.

In this regard, Tinsley et al. [5] reported RMR prediction equations based on FFM and body mass in bodybuilding athletes. The authors observed that RMR values obtained from these equations closely matched those measured by indirect calorimetry. Although their two equations showed good predictive capability, the FFM-based equation produced more accurate results than the BM-based equation. It is worth noting that FFM was determined by dual-energy X-ray absorptiometry (DXA), a precise imaging method often considered a valid laboratory method for FFM analysis. However, DXA may not be applicable in some exercise settings due to its high costs.

In contrast, although bioelectrical impedance analysis (BIA) is widely used in clinical practice due to the lower cost of estimating FFM, it may be less accurate for assessing body composition than DXA. Therefore, it is necessary to determine whether the difference in the estimation of FFM between BIA and DXA would impair the accuracy of the Tinsley et al. [5] equation. Thus, the current study aimed to determine the level of agreement between FFM estimates from BIA and DXA and their use in estimating RMR in men undergoing resistance training. A secondary aim was also to compare the equation that utilizes body mass with the one that uses FFM from DXA.

## 2. Methods

### 2.1. Participants

Thirty healthy college men were selected for this study. All participants had previous experience with resistance training and had been practicing continuously for at least four months, with a minimum frequency of three weekly sessions. Initially, participants completed a detailed health history questionnaire. They were included in the study only if they did not show signs or symptoms of disease, did not use medication, were not using nutritional supplements, did not use androgenic anabolic steroids, did not present any injuries and orthopedic limitations, and were not competitive athletes. The participants signed an informed consent form after being informed about the study’s procedures, objectives, possible benefits, and risks. The study was conducted according to the Declaration of Helsinki and approved by the local Research Ethics Committee.

### 2.2. Anthropometry

Body mass was measured to the nearest 0.1 kg using a calibrated electronic scale (Balmak, Laboratory Equipment Labstore, Curitiba, PR, Brazil), with participants wearing light workout clothing and no shoes. Height was measured to the nearest 0.1 cm by a stadiometer attached to the scale with subjects standing and no shoes. Body mass index (BMI) was calculated as body mass in kilograms divided by the square of height in meters.

### 2.3. Dual-energy X-ray absorptiometry

This study used DXA as a reference method to analyze FFM. Measurements were carried out using a Lunar Prodigy, model NRL 41,990 (GE Lunar, Madison, WI, USA). Before scanning, participants were instructed to remove all objects containing metal. Scans were performed with the participants lying in the supine position along the table’s longitudinal centerline axis. The feet were taped together at the toes to immobilize the legs, while the hands were maintained in a pronated position within the scanning region. Participants remained motionless during the entire scanning procedure. A skilled laboratory technician carried out both calibration and analysis. Equipment calibration followed the manufacturer’s recommendations. The software generated standard lines that set apart the limbs from the trunk and head. These lines were adjusted by the same technician using specific anatomical points determined by the manufacturer. Analyses during the intervention were performed by the same technician who was blinded to group identity throughout the investigation. Body composition measurements were carried out in the morning after an overnight fasting. Room temperature was maintained constant at 19–23ºC for body composition measurements. Participants remained lying for 10 min before each exam for body fluid redistribution.

### 2.4. Bioelectrical impedance analysis

A bioelectrical impedance spectroscopy analyzer (Xitron Hydra, model 4200, Xitron Technologies, San Diego, CA, USA) was used to estimate FFM. Before measurement, participants were instructed to remove all objects containing metal. Measurements were performed on a table isolated from electrical conductors, with participants lying supine along the table's longitudinal centerline axis, legs abducted at an angle of 45º relative to the body midline, and hands pronated. After cleaning the skin with alcohol, two electrodes were placed on the surface of the right hand and two on the right foot by procedures described elsewhere [9]. The spectral bioelectrical impedance analyzer was calibrated daily according to the manufacturer's recommendations. Participants were instructed to urinate ~30 min before the evaluation, refrain from ingesting food or drink during the previous four hours, avoid strenuous physical exercise during the preceding 24 h, refrain from consumption of alcoholic and caffeinated beverages during the preceding 48 h, and avoid the use of diuretics for at least seven days prior each assessment. Body composition assessment was conducted in the morning hours.

From the quantification of Resistance (R) at a frequency of 50 kHz measured by BIA, FFM was calculated using the predictive equation proposed by Matias et al. [10]:

$$\mathrm{FFM}\;=\;2.261\;+\;0.328\;x\;\mathrm{heigh}t^{2}/R\;+\;0.525\;x\;\mathrm{body}\;\mathrm{mass}\;x\;\mathrm{sex}$$

where height was in cm, resistance was in ohm, body mass was in kg, and sex = 1 for men.

### 2.5. Resting metabolic rate

To calculate the RMR, we applied two equations developed by Tinsley et al. [5]. One equation uses FFM (RMR = 25.9 × FFM + 284), while the other utilizes body mass (RMR = 24.8 × body mass + 10).

### 2.6. Statistical analysis

The Shapiro-Wilk test was used to analyze data distribution. Pearson’s correlation coefficient was used to assess the relationship between the methods for estimating the RMR. A simple linear regression was performed to explain FFM and RMR from the reference method and to analyze the standard error of estimation (SEE) and the adjusted R squared. A paired-sample t-test was applied to compare FFM values determined by DXA and BIA. A one-way analysis of variance was used to compare RMR among the methods. Bland-Altman analysis of agreement was used to assess bias and limits of agreement between methods. Statistical significance was set at *p* < 0.05. Data were analyzed using Statistical Package for the Social Sciences (SPSS), version 20.0 (IBM, Armonk, NY, USA).

## 3. Results

**Figure 1.**
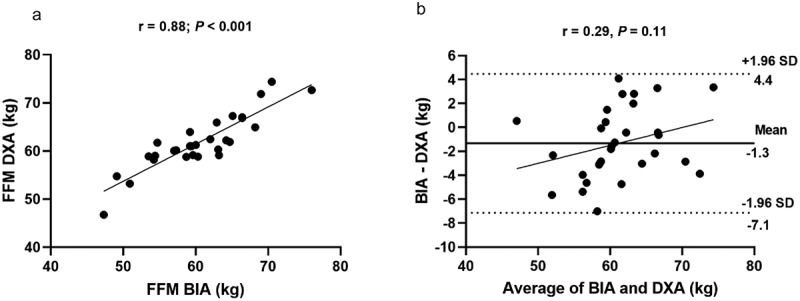
Panel a: Correlation between fat-free mass derived from dual-energy X-ray absorptiometry (DXA) and bioelectrical impedance analysis (BIA). Panel b: Bland-Altman plot between methods for estimating fat-free mass in 30 resistance-trained men. In panel B, the center line represents the mean difference between the methods, and the upper and lower lines represent the 95% limits of agreement (±1.96 SD).

The general characteristics of the sample are described in Table 1. Table 2 presents the FFM values according to the measurement method. FFM estimated by BIA was significantly lower than that measured by DXA (*p* < 0.05). Figure 1 shows the relationship (Panel A) and agreement (Panel B) between methods for measuring FFM. A positive and significant correlation (*p* < 0.05) was observed between DXA and BIA to estimate FFM (y = 15.14 + 0.77×; R^2^ = 0.78; SEE = 2.6 kg); however, it was observed that BIA underestimated FFM compared to DXA with a bias of −1.3 kg.

**Table 1.** General characteristics of the sample (*n* = 30).

Variables	Mean ± SD	95% CI
Age (years)	22.7 ± 4.4	21.0, 24.3
Body mass (kg)	70.0 ± 8.7	66.7, 73.2
Height (cm)	174.6 ± 6.7	172.1, 177.1
BMI (kg/m^2^)	22.9 ± 2.3	22.0, 23.7
Body fat (%)	13.6 ± 6.5	11.2, 15.9
FFMI (kg/m^2^)	20.2 ± 1.4	19.7, 20.7

BMI = Body mass index. FFMI = fat-free mass index.

**Table 2.** Fat-free mass according to method in resistance-trained men (*n* = 30).

Variable	DXA	BIA	Difference DXA – BIA	*P*
	Mean ± SD (95% CI)	Mean ± SD (95% CI)	Mean ± SD (95% CI)	
FFM (kg)	61.7 ± 5.6 (59.6, 63.8)	60.4 ± 6.4 (58.0, 62.8)	1.3 ± 2.9 (0.2, 2.4)	0.02

FFM = Fat-free mass. DXA = dual-energy X-ray absorptiometry. BIA = bioelectrical impedance analysis.

Information related to RMR according to the prediction method is presented in Table 3. No significant difference was observed among the methods used to estimate RMR (*p* > 0.05).

**Table 3.** Resting metabolic rate according to method in resistance-trained men (*n* = 30). Data presented as mean, standard deviation, and 95% confidence interval.

Variable	Fat-free mass*		Body mass*	*P*
	DXA	BIA		
	Mean ± SD (95% CI)	Mean ± SD (95% CI)	Mean ± SD (95% CI)	
RMR (kcal)	1884.2 ± 145.5 (1829.8, 1938.5)	1849.4 ± 167.7 (1786.8, 1912.1)	1746.3 ± 217.3 (1665.1, 1827.4)	0.14

RMR = resting metabolic rate. DXA = dual-energy X-ray absorptiometry. BIA = bioelectrical impedance. BM = body mass. * Tinsley et al. equation.

**Figure 2.**
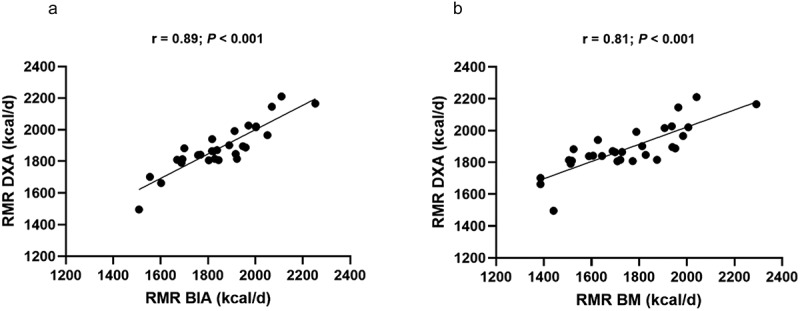
Panel a: Correlation between resting metabolic rate (RMR) calculated from fat-free mass estimated by dual-energy X-ray absorptiometry (DXA) and RMR calculated from fat-free mass estimated by bioelectrical impedance analysis (BIA). Panel b: correlation between the RMR calculated by the fat-free mass estimated by DXA and RMR calculated by the body mass (BM).

**Figure 3.**
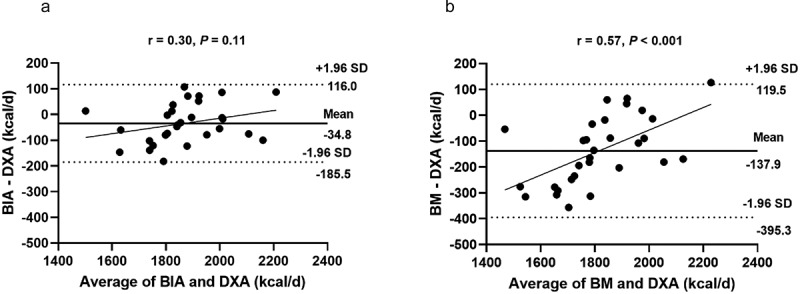
Bland-Altman plot between methods for estimating resting metabolic rate in resistance-trained men (*n* = 30). Panel a indicates the analysis between RMR-DXA and RMR-BIA, and panel B the analysis between RMR-DXA and RMR-BM. The center line represents the mean difference between the methods. The upper and lower lines represent the 95% limits of agreement (±1.96 SD). Fat-free mass-based equation: Resting metabolic rating = 25.9 × fat-free mass + 284. Body mass-based equation: Resting metabolic rating = 24.8 × body mass + 10.

Figure 1 illustrates the relationships between the methods. A positive and significant correlation (*p* < 0.05) was observed between RMR-DXA and RMR-BIA (Panel A) and between RMR-DXA (y = 458.13 + 0.77×; R^2^ = 0.78; SEE = 67.8 kcal) and RMR-BM (y = 938.35 + 0.54×; R^2^ = 0.64; SEE = 87.1 kcal) (Panel B). The mean difference and the limits of agreement between the methods are shown in Figure 2. Compared to the reference method (RMR-DXA), both alternative methods underestimated RMR. However, RMR-BIA displays a lower bias compared to RMR-BM. The absolute limit of agreement for the RMR-BIA was 301.5 kcal, while for the RMR-BM, it was 514.8 kcal (Figure 3).

## 4. Discussion

This study aimed to determine the level of agreement between FFM estimates from BIA and DXA and their use in estimating RMR in men undergoing resistance training. Our findings indicate that utilizing information from DXA or BIA in the Tinsley equation led to similar values of RMR in resistance-trained men. Probabilistic statistics (*P*-value) showed no significant difference between BIA and DXA in calculating RMR. However, it is also important to analyze the individual accuracy. In this sense, Bland-Altman plots are frequently used as an alternative method to compare the differences between the scores of the two methods. This approach can indicate if the bias is low and the limits of agreement are within an acceptable range. In the current study, the bias between BIA and DXA was only −34.8 kcal, suggesting the estimation of RMR using BIA is only slightly underestimated and carries little impact from a practical perspective. The bias in the equation that used FFM from BIA is reduced and represents less than 2% of the resting metabolic rate, thereby having minimal impact from a clinical practice standpoint. However, it is essential to highlight that the absolute limit of agreement was 301.5 kcal.

The amount of FFM can influence prediction since it is a predominant determinant of RMR [11,12]. FFM consists of several organs and tissues, with skeletal muscle mass being the greatest component, comprising 40% to 50% of total body mass, thus substantially impacting daily resting energy [8,13]. Due to the significant metabolic impact of FFM on RMR, several equations based on FFM have been developed to predict RMR [11,12,14–16]. However, individuals who engage in resistance training tend to have higher RMR due to increased skeletal muscle mass. Flack et al. [7] observed an inverse relationship between prediction bias and the magnitude of RMR and FFM, with tested equations becoming less accurate with increasing FFM. This means the magnitude of the underestimation in RMR becomes greater as FFM increases. Therefore, creating a predictive equation for RMR based on FFM for resistance training practitioners seems necessary. The present study supports the utilization of an equation based on FFM to enhance prediction, given that the FFM-based equation showed a smaller bias than the BM-based equation.

The FFM is one of the most frequently monitored parameters to indicate muscular exercise adaptation. Predictive equations for estimating FFM exist for athletes [10] and the general population [17]. The BIA is an affordable and easy-to-use method that has been extensively utilized to measure various components of body composition, such as body fluids, fat mass, FFM, and lean body mass [18,19]. Several predictive equations have been developed considering different populations [18,20]. Some exercisers may present body composition features differently from athletes and untrained individuals [21]. While equations exist for both athletes and non-athletes, these may exclude a significant portion of individuals who train recreationally and may have similar body composition as athletes [21].

Campa et al. [22] reported that athlete-specific and generalized equations could not be used interchangeably when assessing body composition in the general population. It should be noted that certain groups of individuals who engage in regular exercises, such as resistance-trained men, may have body composition characteristics that differ from those of athletes but are still distinctly different from average values in the general population. When determining body composition in resistance-trained exercisers, there appears to be a gap in the available information for individuals who are experienced with resistance training but are not athletes. Campa et al. [22] have shown that the Matias et al. [10] equation adequately predicted FFM in resistance-trained men.

The BIA is a double-indirect method to calculate the FFM, and thus, it has an inherent accumulation of standard errors of estimate. Although our findings indicate that the information from BIA helps estimate RMR, it overestimated FFM by approximately 1 kg. Other studies suggest that some BIA analyzers may need to provide accurate estimations of body composition compared to other standard measurements such as DXA. Thus, care is warranted in selecting the BIA analyzer and the predictive algorithm. The utility of RMR prediction equations based on FFM can be limited, given the additional time, expertise, and expense required to measure FFM using accurate methods, such as DXA.

Moreover, not every FFM-based equation accurately predicts RMR [7]. Therefore, a secondary aim of our study was to compare the Tinsley et al. [5] body mass equation with the one that uses FFM from DXA. RMR prediction equations based on body mass have been used for over a century [6], and numerous distinct equations have been employed [7]. Overall, equations based on body mass tend to underestimate RMR in males by around 400 kcal/day [5]. However, our findings indicated that body mass is a viable alternative to estimate the RMR of resistance-trained individuals with no statistical difference compared to the equation using DXA and a bias of −139.9 kcal.

It should be noted that the agreement between RMR-BM estimates is worse than that observed between RMR-BIA estimates. The data differs on the magnitude of the limits of agreement and the degree of bias. Furthermore, the linear relationship between differences and average (shown in the Bland-Altman plots) indicated a trend for RMR-BM estimates, in which there was a tendency to overestimate the values in individuals with higher RMR and underestimate the values in individuals with lower RMR. The absolute limit of agreement represents 16% of RMR when using FFM from BIA and 27% when using the BM-based equation. This relative magnitude should be considered in a practical scenario. While FFM-based equations seem advantageous due to the reduced bias and smaller limits of agreement, the body mass-based equation is more practical. Our results indicated that the equation based on body mass could accurately predict RMR close to that based on BIA-derived FFM and can be an alternative tool for calculating RMR in the absence of bioimpedance equipment.

Evidence suggests that resistance training may elevate RMR [4]. Thus, RMR prediction equations based on FFM are indicated due to the linear relationship between FFM and RMR. Although age and genetic factors influence RMR, FFM is the primary predictor. Bosselaers et al. [23] found a higher 24-h energy expenditure in resistance-trained individuals than in untrained controls. However, this difference was eliminated after adjusting for differences in FFM. This finding suggests that the high energy expenditure in resistance-trained participants is attributable to their increase in FFM. The rise in RMR induced by resistance training is mainly the result of muscle hypertrophy because resistance training can impact the skeletal muscle mass more than other FFM components, such as internal organs and tissues. However, some experiments have not found a change in RMR in resistance-training practitioners [24,25]. For example, Broeder et al. [24] found no change in RMR in 13 young male volunteers following 12 weeks of high-intensity resistance training despite a 2-kg increase in FFM. Therefore, the small changes in FFM were not sufficient to impact RMR.

Our experiment adopted the DXA as a reference method for measuring FFM. Its technology differentiates the attenuation of photon beams in organic tissues, allowing the construction of images of the area of interest; therefore, it is possible to determine the density and content of bone minerals, the body fat component, and the mass of non-fatty tissues. Hence, it represents a three-compartment method that can be considered valid to estimate FFM and %FM [26–28].

This study has some limitations. Considering that our results are limited to pre-conditioned men, they should not necessarily be extrapolated to another population, such as women, older individuals, or more highly trained persons. Moreover, our sample consisted of recreational resistance-trained men, whereas the Tinsley et al. [5] equation was developed using a sample of bodybuilders. In addition, indirect calorimetry, a gold standard method, was not used for determining RMR values. The lack of ethnic diversity among the participants also may limit the generalization of these results. Also, the information in this study is specific to the BIA analyzer used. Further verification of the current results on different ethnicities and training groups may be required.

## 5. Conclusion

Our results suggest that using FFM derived from DXA or BIA results in similar RMR estimates in young adult men undergoing resistance training. In addition, the equation of Tinsley et al. [5] that uses body mass can be an interesting alternative for estimating RMR when FFM measurements are unavailable.

## References

[1] Murphy C, Koehler K. Energy deficiency impairs resistance training gains in lean mass but not strength: a meta-analysis and meta-regression. Scand J Med Sci Sports. 2022;32(1):125–11. doi: 10.1111/sms.14075 34623696

[2] Churchward-Venne TA, Murphy CH, Longland TM, et al. Role of protein and amino acids in promoting lean mass accretion with resistance exercise and attenuating lean mass loss during energy deficit in humans. Amino Acids. 2013;45(2):231–240. doi: 10.1007/s00726-013-1506-0 23645387

[3] Hall KD, Heymsfield SB, Kemnitz JW, et al. Energy balance and its components: implications for body weight regulation. Am J Clin Nutr. 2012;95(4):989–994. doi: 10.3945/ajcn.112.036350 22434603 PMC3302369

[4] Poehlman ET, Melby C. Resistance training and energy balance. Int J Sport Nutr. 1998;8(2):143–159. doi: 10.1123/ijsn.8.2.143 9637193

[5] Tinsley GM, Graybeal AJ, Moore ML. Resting metabolic rate in muscular physique athletes: validity of existing methods and development of new prediction equations. Appl Physiol Nutr Metab. 2019;44(4):397–406. doi: 10.1139/apnm-2018-0412 30240568

[6] Harris JA, Benedict FG. A biometric study of human basal metabolism. Proc Natl Acad Sci U S A. 1918;4(12):370–373. doi: 10.1073/pnas.4.12.370 16576330 PMC1091498

[7] Flack KD, Siders WA, Johnson L, et al. Cross-validation of resting metabolic rate prediction equations. J Acad Nutr Diet. 2016;116(9):1413–1422. doi: 10.1016/j.jand.2016.03.018 27138231

[8] Gallagher D, Belmonte D, Deurenberg P, et al. Organ-tissue mass measurement allows modeling of REE and metabolically active tissue mass. Am J Physiol. 1998;275(2):E249–58. doi: 10.1152/ajpendo.1998.275.2.E249 9688626

[9] Sardinha LB, Lohman TG, Teixeira PJ, et al. Comparison of air displacement plethysmography with dual-energy X-ray absorptiometry and 3 field methods for estimating body composition in middle-aged men [comparative study]. Am J Clin Nutr. 1998;68(4):786–793. doi: 10.1093/ajcn/68.4.786 9771855

[10] Matias CN, Campa F, Santos DA, et al. Fat-free mass bioelectrical impedance analysis predictive equation for athletes using a 4-compartment model. Int J Sports Med. 2021;42(1):27–32. doi: 10.1055/a-1179-6236 32770535

[11] Nelson KM, Weinsier RL, Long CL, et al. Prediction of resting energy expenditure from fat-free mass and fat mass. Am J Clin Nutr. 1992;56(5):848–856. doi: 10.1093/ajcn/56.5.848 1415003

[12] Cunningham JJ. A reanalysis of the factors influencing basal metabolic rate in normal adults. Am J Clin Nutr. 1980;33(11):2372–2374. doi: 10.1093/ajcn/33.11.2372 7435418

[13] Hayes M, Chustek M, Wang Z, et al. DXA: potential for creating a metabolic map of organ-tissue resting energy expenditure components. Obes Res. 2002;10(10):969–977. doi: 10.1038/oby.2002.132 12376576

[14] ten Haaf T, Weijs PJ, Alemany M. Resting Energy Expenditure Prediction in Recreational Athletes of 18–35 Years: Confirmation of Cunningham Equation and an Improved Weight-Based Alternative. PLoS One. 2014;9(10):e108460. doi: 10.1371/journal.pone.0108460 25275434 PMC4183531

[15] Wang Z, Heshka S, Gallagher D, et al. Resting energy expenditure-fat-free mass relationship: new insights provided by body composition modeling. Am J Physiol Endocrinol Metab. 2000;279(3):E539–45. doi: 10.1152/ajpendo.2000.279.3.E539 10950820

[16] Mifflin MD, St Jeor ST, Hill LA, et al. A new predictive equation for resting energy expenditure in healthy individuals. Am J Clin Nutr. 1990;51(2):241–247. doi: 10.1093/ajcn/51.2.241 2305711

[17] Sun SS, Chumlea WC, Heymsfield SB, et al. Development of bioelectrical impedance analysis prediction equations for body composition with the use of a multicomponent model for use in epidemiologic surveys. Am J Clin Nutr. 2003;77(2):331–340. doi: 10.1093/ajcn/77.2.331 12540391

[18] Campa F, Toselli S, Mazzilli M, et al. Assessment of body composition in athletes: a narrative review of available methods with special reference to quantitative and qualitative bioimpedance analysis. Nutrients. 2021;13(5):1620. doi: 10.3390/nu13051620 34065984 PMC8150618

[19] Lukaski H, Raymond-Pope CJ. New frontiers of body composition in sport. Int J Sports Med. 2021;42(7):588–601. doi: 10.1055/a-1373-5881 33621995 PMC8421000

[20] Coratella G, Campa F, Matias CN, et al. Generalized bioelectric impedance-based equations underestimate body fluids in athletes. Scand J Med Sci Sports. 2021;31(11):2123–2132. doi: 10.1111/sms.14033 34383339 PMC9292858

[21] Campa F, Coratella G. Athlete or non-athlete? This is the question in body composition. Front Physiol. 2021;12:814572. doi: 10.3389/fphys.2021.814572 34975550 PMC8718693

[22] Campa F, Matias CN, Teixeira FJ, et al. Comparison of generalized and athletic bioimpedance-based predictive equations for estimating fat-free mass in resistance-trained exercisers. Nutrition. 2022;102:111694. doi: 10.1016/j.nut.2022.111694 35810579

[23] Bosselaers I, Buemann B, Victor OJ, et al. Twenty-four-hour energy expenditure and substrate utilization in body builders. Am J Clin Nutr. 1994;59(1):10–12. doi: 10.1093/ajcn/59.1.10 8279388

[24] Broeder CE, Burrhus KA, Svanevik LS, et al. The effects of either high-intensity resistance or endurance training on resting metabolic rate. Am J Clin Nutr. 1992;55(4):802–810. doi: 10.1093/ajcn/55.4.802 1550062

[25] Van Etten LM, Westerterp KR, Verstappen FT. Effect of weight-training on energy expenditure and substrate utilization during sleep. Med Sci Sports Exerc. 1995;27(2):188–193. doi: 10.1249/00005768-199502000-00007 7723641

[26] Prior BM, Cureton KJ, Modlesky CM, et al. In vivo validation of whole body composition estimates from dual-energy X-ray absorptiometry. J Appl Physiol. 1997;83(2):623–630. doi: 10.1152/jappl.1997.83.2.623 9262461

[27] Blue MNM, Tinsley GM, Ryan ED, et al. Validity of body-composition methods across racial and ethnic populations. Adv Nutr. 2021;12(5):1854–1862. doi: 10.1093/advances/nmab016 33684215 PMC8528114

[28] Blue MNM, Hirsch KR, Brewer GJ, et al. The validation of contemporary body composition methods in various races and ethnicities. Br J Nutr. 2022:1–11. doi: 10.1017/S0007114522000368 35109945

